# Does the type of abortion provider influence contraceptive uptake after abortion? An analysis of longitudinal data from 64 health facilities in Ghana

**DOI:** 10.1186/s12889-015-1875-2

**Published:** 2015-06-24

**Authors:** Lauren Maxwell, Gertrude Voetagbe, Mary Paul, Alice Mark

**Affiliations:** Institute for Health and Social Policy, McGill University, 1130 Pine Ave West, Montréal, QC H3A 1A3 Canada; Ipas, P.O. Box 9990, Chapel Hill, NC 27515 USA; Ipas Ghana, No. 8 Akosombo Road, Airport Residential Area, Accra, Ghana

**Keywords:** Postabortion contraception, Abortion, Ghana, Midwives

## Abstract

**Background:**

Understanding what factors influence the receipt of postabortion contraception can help improve comprehensive abortion care services. The abortion visit is an ideal time to reach women at the highest risk of unintended pregnancy with the most effective contraceptive methods. The objectives of this study were to estimate the relationship between the type of abortion provider (consultant physician, house officer, or midwife) and two separate outcomes: (1) the likelihood of adopting postabortion contraception; (2) postabortion contraceptors’ likelihood of receiving a long-acting and permanent versus a short-acting contraceptive method.

**Methods:**

We used retrospective cohort data collected from 64 health facilities in three regions of Ghana. The dataset includes information on all abortion procedures conducted between 1 January 2008 and 31 December 2010 at each health facility. We used fixed effect Poisson regression to model the associations of interest.

**Results:**

More than half (65 %) of the 29,056 abortion clients received some form of contraception. When midwives performed the abortion, women were more likely to receive postabortion contraception compared to house officers (RR: 1.18; 95 % CI: 1.13, 1.24) or physicians (RR: 1.21; 95 % CI: 1.18, 1.25), after controlling for facility-level variation and client-level factors. Compared to women seen by house officers, abortion clients seen by midwives and physicians were more likely to receive a long-acting and permanent rather than a short-acting contraceptive method (RR: 1.46; 95 % CI: 1.23, 1.73; RR: 1.58; 95 % CI: 1.37, 1.83, respectively). Younger women were less likely to receive contraception than older women irrespective of provider type and indication for the abortion (induced or PAC).

**Conclusions:**

When comparing consultant physicians, house officers, and midwives, the type of abortion provider is associated with whether women receive postabortion contraception and with whether abortion clients receive a long-acting and permanent or a short-acting method. New strategies are needed to ensure that women seen by physicians and house officers can access postabortion contraception and to ensure that women seen by house officers have access to long-acting and permanent contraceptive methods.

**Electronic supplementary material:**

The online version of this article (doi:10.1186/s12889-015-1875-2) contains supplementary material, which is available to authorized users.

## Background

In Ghana, over one third of women of reproductive age have an unmet need for contraception [1]. Complications of induced abortion are the leading cause of maternal mortality for girls aged 19 and younger and the second leading cause of maternal mortality for women of reproductive age, accounting for an estimated 11 % of maternal deaths [2]. While Ghana has a relatively liberal abortion law [3], a mixture of stigma, lack of knowledge about the law, and limited access to health facilities may explain why unsafe abortion continues to be an important cause of maternal mortality and morbidity [4–6]. Women who seek unsafe abortion often present to health facilities needing uterine evacuation for incomplete abortion, known as postabortion care (PAC).

Providing contraceptive access during an abortion visit has the potential to prevent future unintended pregnancies in a vulnerable population. The abortion visit is an ideal time to offer contraception as the woman may be motivated to use a method, she is known not to be pregnant, and she is in contact with a reproductive health care provider. All methods of contraception are safe to use immediately after an uncomplicated first or second trimester abortion including long-acting and permanent methods (LA/PM) [7–9]. Research that explores determinants of contraceptive uptake for youth is important given that, in Ghana, youth are both more likely to experience an unintended pregnancy and to report having an unsafe abortion [10].

Contraceptive research in Ghana indicates that client and provider factors are important determinants of whether women will accept contraception and their choice of contraceptive method [5, 6, 11, 12]. Variations in demographic factors including region, age, education, parity, and socioeconomic status may also affect contraceptive use [1, 13–15]. Several smaller qualitative studies in Ghana suggest that provider-level factors like sex, age, and provider attitudes around contraception are associated with women’s receipt of contraception [5, 16, 17].

Our study estimates the relationship between the type of abortion provider (consultant physician, house officer, or midwife) and whether women receive postabortion contraception at 64 Ghanaian health facilities. Understanding the relationship between the type of abortion provider and abortion client’s receipt of contraception can help design interventions to better reach the women at the highest risk of repeat unintended pregnancy with the most effective methods.

## Methods

This is a retrospective cohort study that includes all abortion procedures conducted between January 1, 2008 and December 31, 2010 at 64 health care facilities in the Ashanti, Eastern, and Greater Accra regions of Ghana. The facilities included 23 public hospitals, 37 public health centers, and 4 private maternity homes which are small clinics where midwives provide reproductive health services. All facilities had partnered with Ipas, a global, non-governmental organization that advocates for women’s sexual and reproductive health and rights. The collaboration with Ipas was part of facilities’ participation in the Reducing Maternal Mortality and Morbidity (R3M) program, a Ghana Health Services program designed to improve comprehensive abortion care services [18]. Consultant physicians are physicians of varying levels of seniority who have received their Bachelor of Medicine and Bachelor of Surgery degree. House officers are pre-practice physicians who have graduated from medical school during the last year and who are trained in uterine evacuation and contraceptive counseling during their 6 month obstetrics and gynecology rotation. Midwives are either nurses who receive a year of midwifery training following 2 years of nursing school or community health officers or college graduates who receive 2 years of midwifery training. Prior to and during the study period, Ipas staff worked in collaboration with the Ghana Health Service to train abortion providers in comprehensive abortion care and visited partner facilities to monitor the quality of abortion services and to ensure abortion providers’ routine completion of facility logbooks provided through the R3M program.

### Data

During the study period, 29,463 procedures were recorded in facility logbooks. Providers entered abortion case data into logbooks provided by the R3M program; data were later entered into a database by Ipas program staff. Ipas received permission from individual health facilities and from the Ghana Health Service to collect and analyze logbook data. The logbook captures demographic variables (client age), indication (induced or PAC), type of procedure (manual vacuum aspiration, electric vacuum aspiration, uterine evacuation with misoprostol alone or mifepristone and misoprostol, dilation and curettage, or extraction) and type of postabortion contraception. Short-acting (condoms, oral contraceptives, injectables) and LA/PM (intrauterine devices, contraceptive implants, and sterilization) are available at all facility levels with the exception of sterilization which is not offered at the midwife-run maternity homes. We excluded listings for 368 abortion procedures that were referred to alternate facilities and 39 procedures that were listed as molar or ectopic pregnancies or that were classified as intrauterine fetal demise.

### Statistical analysis

The outcomes of interest were whether women received postabortion contraception or not and, for women who received contraception, whether they received a short or LA/PM. Because odds ratios estimated using logistic regression are known to overestimate relative risks for prevalent outcomes [19], we used Poisson regression with robust standard errors to estimate 1) the relative risk of receipt of postabortion contraception; and 2) the relative risk (RR) of receiving a LA/PM rather than a short-acting method by provider type. We compared covariates (four age categories, trimester, indication for abortion, uterine evacuation procedure type, facility level, and facility region) between women who did or did not receive contraception with bivariate Poisson regression models.

We constructed a directed acyclic graph to identify probable confounders based on our review of the literature and understanding of the causal relationship between provider type and women’s adoption of contraception. Women who go to the same clinic often receive the same contraceptives because of clinic-specific protocols, organization, and supplies. To eliminate the possibility of obtaining estimates that are biased by differences across facilities, we included a fixed effect for each health facility in the adjusted models. The health facility fixed effects control for all measured and unmeasured differences across facilities [20].

We identified age and indication for abortion service (induced abortion or PAC) *a priori* as potential effect measure modifiers [21]. Likelihood ratio tests were used to evaluate the predictive value of the predefined interaction terms (at α = 0.05) between age and type of provider, indication for abortion service and type of provider, and age and indication for abortion service. We used the modified STROBE guidelines to present the results from the interaction between client age and abortion indication [22]. We used chi-squared tests to compare the probability of the outcome for abortion cases missing information on client and/or trimester and cases that had no missing information. Cases with missing information on client age and/or trimester were as likely as included cases to have received postabortion contraception (α = 0.05) and were excluded from the multivariate regression analysis (*n* = 2931 or 10 % of abortion cases). The multivariate analysis was based on data from 26,125 abortion procedures. All analysis was conducted in Stata SE version 13 (College Station, TX: StataCorp LP). The research protocol was approved by the Research Ethics and Compliance Institutional Review Board at McGill University, Montréal, Canada. The McGill University Institutional Review Board functions in accordance with the Tri-Council Policy Statement and the U.S. Code of Federal Regulations, guidelines that are also used by Ghanaian IRBs [23]. The authors were not able to identify any national regulations or guidelines for human subjects research in Ghana. As the dataset did not contain any information that could be used to identify individual participants or providers we did not seek additional approval from a Ghanaian Ethics Committee for this analysis.

## Results

### Baseline characteristics

Of the 29,056 procedures included in the initial dataset, 65 % of women were recorded as having received postabortion contraception. Fifteen percent of women received a LA/PM while 50 % received a short-acting method of contraception (Table 1). Most abortion services were provided at hospitals (80 %). Midwives and physicians provided a comparable number of services (43 % and 47 % respectively) while house officers were responsible for 10 % of services. Forty-one percent of procedures were induced abortions and 59 % were PAC. Most PAC procedures (61 %) were performed in hospitals, health centers and private maternity homes accounted for 26 % and 13 % of PAC procedures, respectively (data not shown). In hospitals, midwives performed 12 % of PAC and 67 % of induced procedures, while physicians and house officers provided 70 % and 18 % of PAC procedures and 32 % and <1 % of induced procedures (data not shown).

**Table 1:** Facility and client-level characteristics of abortion clients (*n* = 29,056)

Variable	N	(%)
***Facility-level characteristics***		
Women served by facility-level		
Hospital	23,261	(80.1)
Health center	4,141	(14.3)
Maternity home	1,654	(5.7)
Women seen by type of provider		
House Officer	2,799	(9.6)
Physician	13,753	(47.3)
Midwife	12,504	(43.0)
Women served by facility region		
Ashanti	6,476	(22.3)
Eastern	8,777	(30.2)
Greater Accra	13,803	(47.5)
***Client-level characteristics***		
Client age (years)		
10-19	4,400	(15.4)
20-29	15,321	(52.7)
30-39	7,451	(25.6)
40-49	1,117	(3.8)
Missing	767	(2.6)
Mean client age (SD), years	26	(6.7)
Trimester		
1st Trimester	24,077	(82.9)
2nd Trimester	2,509	(8.6)
Missing	2,470	(8.5)
Mean gestational age (SD), weeks	8.6	4.2
Uterine evacuation procedure type		
MVA or EVA	27,870	(95.9)
Medical abortion^a^	1,083	(3.7)
D&C or D&E	103	(0.4)
Indication for abortion		
Postabortion care	17,019	(58.6)
Induced abortion	12,037	(41.4)
Postabortion contraception received		
Short-acting method^b^	14,521	(50.0)
Long-acting and permanent method^c^	4,240	(14.6)
No method	10,295	(35.4)
Type of postabortion contraception		
Male condom	2,680	(9.2)
Female condom	668	(2.3)
Oral contraceptives	4,550	(15.7)
Injection	6,553	(22.6)
Spermicide/Emergency contraception	70	(0.2)
IUD	1,347	(4.6)
Contraceptive implant	2,799	(9.6)
Bilateral tubal ligation	94	(0.3)
No method	10,295	(35.4)

*MVA*, manual vacuum aspiration; *EVA*, electric vacuum aspiration; *D&C*, dilation & curettage; *D&E*, dilation & extraction; *IUD*, intra-uterine device

^a^Medical abortion refers to treatment with either misoprostol or a combination misoprostol and mifepristone

^b^Short-acting contraceptive method refers to: barrier methods, oral contraception, injectables, spermicide, emergency contraception

^c^Long-acting and permanent contraceptive method refers to: IUD, contraceptive implant, bilateral tubal ligation

All covariates were significantly associated with abortion clients’ receipt of contraception in unadjusted bivariate analysis (Table 2). Women who received an induced abortion were more likely to receive contraception than women who received PAC (RR: 1.55; 95 % CI: 1.52, 1.57). Women who had a second trimester procedure were less likely to receive contraception than first trimester clients (RR: 1.25; 95 % CI: 0.72, 0.78).

**Table 2:** Demographic and clinical care factors associated with uptake of contraception among abortion clients (*n* = 29,056)

Variable	Received postabortion contraception *n* = 18,761 %	Did not receive postabortion contraception *n* = 10,295 %	Univariate Poisson regression RR (95 % CI)	P-value^*^
Facility-level				
Hospital	59.4	40.6	1	
Health center	82.9	17.1	1.39 (1.37, 1.42)	
Maternity home	90.9	9.1	1.53 (1.50, 1.56)	<0.001
Facility region				
Ashanti	66.2	33.9	1	
Eastern	80.2	19.8	1.21 (1.19, 1.24)	
Greater Accra	53.9	46.1	0.81 (0.80, 0.83)	<0.001
Type of provider				
House Officer	45.8	54.2	1	
Physician	53.2	46.8	1.16 (1.11, 1.21)	
Midwife	81.2	18.8	1.77 (1.70, 1.85)	<0.001
Client age				
10-19	72.9	27.1	1	
20-29	66.2	33.8	0.91 (0.89, 0.93)	
30-39	60.3	39.7	0.83 (0.81, 0.85)	
40-49	60.2	39.8	0.82 (0.78, 0.87)	<0.001
Trimester				
1st Tri	67.9	32.1	1	
2nd Tri	50.8	49.2	0.75 (0.72, 0.78)	<0.001
Procedure type				
MVA or EVA	63.9	36.1	1	
Medical abortion^b^	79.9	20.1	1.25 (1.21, 1.29)	
D&C or D&E	76.7	23.3	1.20 (1.08, 1.33)	<0.001
Indication for abortion				
Postabortion care	52.6	47.4	1	
Induced abortion	81.5	18.6	1.55 (1.52, 1.57)	<0.001

*RR*, risk ratio; *CI*, confidence interval; *MVA*, manual vacuum aspiration; *EVA*, electric vacuum aspiration; *D&C*, dilation & curettage; *D&E*, dilation & extraction

^*^P-value for the 2-tailed test of the null hypothesis that the regression coefficient is equal to zero

^b^Medical abortion refers to treatment with either misoprostol or a combination misoprostol and mifepristone

### Type of provider and receipt of postabortion contraception

After controlling for differences across facilities, provider type, client age, trimester, and indication for abortion were significantly associated with the receipt of a postabortion contraception method and were included in the final models. Potential interaction effects between provider type and client age and provider type and abortion indication (PAC or induced) were not statistically significant. We found a statistically significant interaction between client age and abortion indication when estimating the association between provider type and whether women received postabortion contraception. The interaction between client age and abortion indication was not statistically significant when estimating whether women received a short-acting rather than a LA/PM.

Women seen by midwives were more likely to receive a contraceptive method than women seen by house officers (RR: 1.18; 95 % CI: 1.13, 1.24; Table 3) or women seen by consultant physicians (RR: 1.21; 95 % CI: 1.18, 1.25), after adjusting for women’s age, trimester, indication for abortion service, the interaction between age and indication for abortion service, and differences across facilities. In contrast, women seen by consultant physicians were as likely to have received a contraceptive method as those seen by house officers (RR: 0.97; 95 % CI: 0.93, 1.02). The type of abortion provider was associated with whether women received a short-acting versus a LA/PM. Compared to women seen by house officers, women seen by midwives and by consultant physicians were more likely to receive a LA/PM rather than a short-acting contraceptive method (RR: 1.46; 95 % CI: 1.23, 1.73; RR: 1.58; 95 % CI: 1.37, 1.83, respectively).

**Table 3:** Poisson regression estimates of main and interaction effects for postabortion contraceptive uptake and uptake of a long-acting and permanent rather than a short-acting contraceptive method

	Receipt of any postabortion contraception (*n* = 26,125)			Receipt of LA/PM for women who receive postabortion contraception (*n* = 17,456)
	Main effects	Interaction effects		
	Uptake of any contraception	Uptake of any contraception by induced clients	Uptake of any contraception by PAC clients	
	RR (95 % CI)	RR (95 % CI)	RR (95 % CI)	RR (95 % CI)
Type of provider				
House Officer	1			1
Physician	0.98 (0.93, 1.02)			1.58 (1.37, 1.83)
Midwife	1.18 (1.13, 1.24)			1.46 (1.23, 1.73)
Age category				
10-19		1	1	1
20-29		1.05 (1.03, 1.07)	0.92 (0.88, 0.95)	1.05 (0.97, 1.13)
30-39		1.11 (1.08, 1.13)	0.87 (0.83, 0.90)	1.52 (1.41, 1.64)
40-49		1.10 (1.05, 1.16)	0.87 (0.81, 0.95)	1.96 (1.75, 2.20)
Trimester				
1st tri	1			1
2nd tri	0.98 (0.94, 1.02)			1.07 (0.96, 1.73)
Indication for abortion				
Postabortion care				1
Induced abortion				1.35 (1.24, 1.46)

*LA/PM*, long-acting and permanent method; *RR*, risk ratio; *CI*, confidence interval

All models include a facility fixed effect, an interaction term between client age category and indication for abortion (induced or PAC), and are adjusted for provider type, client age category, trimester, and indication for abortion

We include columns for induced and PAC procedures for the estimated RR for each age category because the association between age and receipt of contraception is modified by whether the procedure is considered a PAC or an induced procedure ([see Additional file 1] for additional analysis of the interaction between age category and abortion indication)

### Interaction between client age and abortion indication (induced or PAC)

**Fig. 1:**
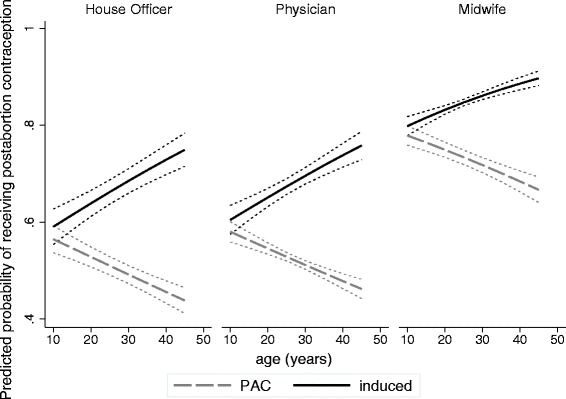
Difference in the probability of postabortion contraceptive uptake by client age and indication for the abortion (induced or PAC) for each type of provider (house officer, physician, midwife). All models include a facility fixed effect, an interaction term between age (continuous) and indication for abortion (induced or PAC), and are adjusted for provider type, client age (continuous) trimester, and indication for abortion

As shown in Fig. 1, after adjusting for trimester and including fixed effects to control for facility-level variation, abortion indication modified the association between client age and receipt of postabortion contraception similarly for each type of provider. While induced clients were more likely to receive contraception than PAC clients at all ages, the effect of abortion indication on contraceptive uptake was strongest for the oldest clients (See Additional file 1). Girls aged 10–19 who had an induced abortion were more likely to receive contraception than girls of the same age who had received PAC (RR: 1.07; 95 % CI: 1.03, 1.11). Women aged 20–29 and women aged 30–39 with induced abortions were more likely to receive postabortion contraception compared to women of the same age who had had a PAC procedure (RR: 1.23; 95 % CI: 1.19, 1.26; RR: 1.37; 95 % CI: 1.32, 1.42, respectively).

### Additional covariates and the receipt of postabortion contraception

Age was significantly associated with the receipt of a LA/PM versus a short-acting contraceptive method. Compared to girls ages 10–19, women ages 30–39 and women ages 40–49 were more likely to receive a LA/PM versus a short-acting method (RR: 1.52; 95 % CI: 1.41, 1.64; RR: 1.96; 95 % CI: 1.75, 2.20, respectively). Indication for the abortion (induced or PAC) was associated with the type of method adopted; women who had an induced abortion were more likely to receive a LA/PM rather than a short-acting contraceptive method when compared to women who received PAC (RR: 1.35; 95 % CI: 1.24, 1.47). While in bivariate analysis, women presenting for 2nd trimester abortions were 25 % less likely to receive contraception than women with 1st trimester abortions, the association between trimester and receipt of contraception was not significant in the multivariate analysis because 2nd trimester cases are more likely to receive PAC (91 % of 2nd trimester clients received PAC compared to 54 % of 1st trimester clients) and having received PAC was more predictive of women not receiving postabortion contraception than trimester.

## Discussion

Our study found a strong association between the type of abortion provider and whether women receive postabortion contraception. Women seen by midwives were 18 % more likely to receive postabortion contraception than women seen by house officers (95 % CI: 13 %, 24 %) and 21 % more likely than women seen by consultant physicians (95 % CI: 18 %, 25 %) after controlling for abortion indication, client age, gestational age, and facility-level heterogeneity. Compared to women seen by house officers, contraceptors seen by consultant physicians were 58 % (95 % CI: 37 %, 83 %) and those seen by midwives were 46 % (95 % CI: 23 %, 73 %) more likely to receive a LA/PM.

We found evidence that the association between age and receipt of postabortion contraception varies by the indication for abortion (induced or PAC). As age increased, PAC clients were less and induced clients were more likely to receive postabortion contraception. For PAC clients, the inverse relationship between age and the likelihood of receiving postabortion contraception may be explained in part because older PAC patients are more likely to be treated for miscarriage of wanted pregnancies and may have been more likely to refuse contraception than younger PAC clients presenting after unsafe abortion. Young women who received induced abortions were significantly less likely to receive contraception when compared to older women. This finding mirrors that of a recent study in Kenya which found that younger abortion clients were more likely to refuse contraception than older clients because they were afraid of infertility and because of lack of knowledge of available contraceptive methods [21]. Although the reasons for young women’s reduced uptake of postabortion contraception are not measured in our study, removing barriers to access and addressing common misconceptions about long-acting and reversible contraception is important for this vulnerable population.

Our results suggest that considering the indication for abortion (PAC or induced) is important for understanding the association between age and receipt of postabortion contraception. Given that the effect of abortion indication on the association between age and receipt of contraception acts in opposite directions of equal magnitude, age is not a significant predictor of receipt of postabortion contraception in models that fail to account for the interaction. In contrast, by accounting for the interaction, we found that age is strongly associated with whether or not women receive postabortion contraception.

### Strengths & limitations of the study

This analysis has a number of strengths. With almost 30,000 cases from over 60 facilities, we were able to look at multiple factors associated with postabortion contraception, control for facility-level variation and client-level confounding, and evaluate the potential for effect measure modification. Prior quantitative studies that have addressed predictors of postabortion contraception have not included a large enough sample of induced abortions to fully explore the modification of the effect of age by abortion indication.

The main limitation in this analysis is that factors that were not measured in this dataset related to the provider (sex), procedure (contraceptive counseling, whether the procedure was inpatient or outpatient), or the patient (socioeconomic status, marital status, parity, contraceptive intentions, future pregnancy intention) may have affected the outcome. As mentioned previously, some PAC cases are the result of pregnancy failure rather than incomplete abortion after unsafe abortion, which decreases the number of women who may want contraception after a PAC procedure. The proportion of PAC cases that are spontaneous abortions is unknown for the entire sample and likely varies by facility level. Because pregnancy failure is most likely to occur prior to the 13th week of gestation [24] and is more likely in women aged 35–50 [25] we expect that older women and women at earlier gestational ages would be more likely to have sought PAC after a spontaneous abortion than younger women or women with higher gestational ages. Without knowing the distribution of pregnancies by maternal age and gestational age in the source population, we are unable to estimate the proportion of PAC clients in our sample who presented following spontaneous rather than induced abortions. In a recent study of PAC clients at Komfo Anokye Teaching Hospital, a tertiary institution in Ghana, 71 % of PAC clients reported presenting for services following a self-induced abortion which suggests that the rate of self-induced or unsafe abortion among PAC clients in our sample is likely to be high [26].

The 60 facilities considered in the adjusted analysis are mainly located in urban areas. Other studies of contraceptive behavior in Ghana suggest that women in rural areas less likely to use contraception than women in urban areas and a recent study indicated that providers at health facilities that participated in the R3M program are more likely to provide safe abortion than providers at facilities that did not participate [18] which limits the generalizability of our findings.

We used facility fixed effects to ensure that estimates were not affected by time-fixed facility-level differences like contraceptive supply and prescribing practices. One downside of the fixed effects approach is that we cannot estimate the association between provider type and women’s adoption of contraception in facilities that only have one type of provider, because, for these facilities, provider type is a fixed at the facility level. Therefore, in multivariate regressions where we used facility fixed effects, we excluded procedures conducted at the four private maternity homes that were only staffed by midwives. Similarly, in the fixed effects models, we were not able to examine the association between variables whose value was fixed at the facility level [e.g. region; type of facility (hospital, health center, or maternity home)] and contraceptive uptake. Frequently, in modelling, we are faced with a tradeoff between bias and variance. In this case, we chose to reduce the level of bias by eliminating facility-level variation. The tradeoff for this reduction in bias was that we could no longer include women seen at private maternity homes in the fixed effects models; we were not able to use our entire sample, but our estimates are not biased by facility-level differences.

### Implications for practice

Our finding that women seen by midwives are more likely to receive postabortion contraception than women seen by other types of providers is in keeping with prior studies that have highlighted midwives’ skill in providing comprehensive abortion and postabortion care [27, 28]. Women seen by house officers were significantly less likely to receive postabortion contraception than those seen by midwives and were significantly more likely to receive a short-acting method than a LA/PM when compared to women seen by midwives and physicians. In our study, 1-in-10 abortion services were provided by house officers. Because house officers have graduated from medical school within the last year, pre-service or on-the-job training in LA/PMs may help support their skills. Finally, because house officers and physicians treated more postabortion care cases than midwives, concerns with complications may have limited IUD use. The finding that use of implants and IUDs was lower for house officers than for physicians, despite the fact that they can be used even after complicated PAC cases, may show that provider factors still play a role in LA/PM provision.

In Ghana, younger women are at the highest risk of unintended pregnancy [10]. Our study indicates that young women may face barriers to accessing postabortion contraception. Long-acting and reversible contraceptive methods are effective in reducing unintended pregnancies and have been shown to reduce future unintended pregnancies and repeat abortions among abortion clients [29, 30]. Encouraging providers to counsel youth about all methods, including LA/PMs, could help to reduce unintended pregnancy within this high-risk population. Our analysis showed that women who received induced services were more likely to receive a LA/PM compared to women who received PAC care. Providers should understand that all contraceptive methods, including LA/PMs, are safe for women who need uncomplicated PAC services and choose to use contraception [7].

The organization of service delivery within facilities may be related to the low levels of contraceptive uptake among women seen by physicians and house officers and among PAC clients. In Ghana, the majority of PAC clients are sent to the inpatient gynecology ward where they receive services from house officers and physicians and are referred to family planning units for contraceptive care. In contrast, midwives are more likely to provide PAC service and contraceptive counseling at the same time in the same outpatient location. Prior research indicates that PAC clients are most likely to receive contraception when contraceptive counseling and supplies are provided at the time of abortion service [31–33]. Women with uncomplicated PAC (bleeding but no infection, perforation, hemorrhage) may be better seen by midwives as outpatients rather than on the ward.

## Conclusions

While several studies have indicated that there is a high level of awareness of modern contraceptive methods among Ghanaian women [4, 5], a significant percentage of Ghanaian women continue to report an unmet need for contraception and over 30 % of births are mistimed or unintended [1]. This research highlights the central role that midwives can play in reaching women at the highest risk of unintended pregnancy with contraception. The analysis also indicates a need to collaborate with physicians and house officers to ensure that they provide women with contraception and to increase house officer’s provision of LA/PM. Ensuring that postabortion care and contraceptive services are provided in the same location, training midwives to serve PAC clients, and working with all providers to improve young women’s access to contraception are important interventions to ensure that abortion clients can access postabortion contraception.

## Additional files

Additional file 1:
**Additional information on the modification of the association between age and contraceptive uptake by indication for the abortion (induced or PAC).**
