# Supplementary material for: Does the type of abortion provider influence contraceptive uptake after abortion? An analysis of longitudinal data from 64 health facilities in Ghana
Source: BMC Public Health. 2015 Jun 24;15:586. doi: 10.1186/s12889-015-1875-2 (PMC4478624; doi:10.1186/s12889-015-1875-2)
Supplement: Additional file 1: — Additional information on the modification of the association between age and contraceptive uptake by indication for the abortion (induced or PAC). [file 12889_2015_1875_MOESM1_ESM.pdf]

Additional file 1: **Additional information on the modification of the association between age and contraceptive uptake by indication for the abortion (induced or PAC)**

In keeping with the revised STROBE guidelines for the presentation of effect measure modification [1], we present the effect estimates for each potential combination of age category and indication for abortion in Tables S1 and S2. We have included this additional information in the Appendix because we presented a graphical representation of the modification of the association between age and contraceptive uptake by indication for the abortion (induced or PAC) in the text (Figure 1).

Table S1. Modification of the association between age and contraceptive uptake by indication for the abortion (induced or PAC).

	Abortion Indication				
	PAC		Induced		
Age category	N with/without contraception	RR (95% CI)	N with/without contraception	RR (95% CI)	RR (95% CI) for induced vs PAC within age categories
10-19	1,156/722	1.0	2,053/469	1.0	1.07 (1.03, 1.11)
20-29	4,873/3,936	0.92 (0.88, 0.95)	5,273/1,239	1.05 (1.03, 1.07)	1.23 (1.19, 1.26)
30-39	2,423/2,529	0.87 (0.83, 0.90)	2,072/427	1.11 (1.08, 1.13)	1.37 (1.32, 1.42)
40-49	331/368	0.87 (0.81, 0.95)	341/77	1.10 (1.05, 1.16)	1.35 (1.23, 1.47)

RR=risk ratio; CI=confidence interval
All models include a facility fixed effects, an interaction term between client age category and indication for abortion (induced or PAC), and are adjusted for provider type, client age category, trimester, and indication for abortion.

Table S2. Departure from additivity of effects on the relative risk scale and multiplicativity of effects on the multiplicative scale, given the modification of the association between age and contraceptive uptake by indication for the abortion (induced or PAC).

Age category	Measure of interaction on the additive scale comparing each age category to clients aged 10-19 for induced vs PAC clients<br>RERI (95% CI)	Measure of interaction on the multiplicative scale comparing each age category to clients aged 10-19 for induced vs PAC clients<br>Ratio of RRs (95% CI)
20-29	0.14 (0.10, 0.18)	1.15 (1.10, 1.20)
30-39	0.25 (0.20, 0.29)	1.28 (1.22, 1.34)
40-49	0.23 (0.14, 0.32)	1.26 (1.14, 1.38)

PAC=postabortion care; RERI=relative excess risk due to interaction; CI=confidence interval; RR=risk ratio
All models include a facility fixed effects, an interaction term between client age category and indication for abortion (induced or PAC), and are adjusted for provider type, client age category, trimester, and indication for abortion.

The relative excess risk due to interaction (RERI) estimates the departure from additivity of effects on the relative risk scale [2]. Departures from additivity are considered a more important for assessing the public health importance of interactions [1]. The RERI of 0.23 (95% CI: 0.14, 0.32) suggests that, after adjusting for provider type, trimester, and differences across facilities, there is some indication that the estimated joint effect of the indication for abortion (induced vs. PAC) and client age category (ages 40-49 vs. ages 10-19) was greater than the sum of the estimated effects of indication for abortion and client age. In adjusted models, the measure of interaction on the multiplicative scale (the ratio of the RRs), comparing the likelihood of adopting contraception for induced vs. PAC procedures for women aged 40-49 to those aged 10-19 was 1.26 (95% CI: 1.14, 1.38) which suggests that the estimated joint effect of the indication for the procedure (induced vs. PAC) and the contrast of women aged 40-49 vs. aged 10-19 was slightly greater than the product of the estimated effects of each of these exposures alone which indicates the presence of positive interaction on the multiplicative scale.

REFERENCES

1. Knol MJ, VanderWeele TJ: **Recommendations for presenting analyses of effect modification and interaction**. *International Journal of Epidemiology* 2012, **41**(2):514-520.
2. Richardson DB, Kaufman JS: **Estimation of the Relative Excess Risk Due to Interaction and Associated Confidence Bounds**. *American Journal of Epidemiology* 2009, **169**(6):756-760.
